# Recent advances in understanding and managing aortic stenosis

**DOI:** 10.12688/f1000research.11906.1

**Published:** 2018-01-16

**Authors:** Mathias Van Hemelrijck, Maurizio Taramasso, Carlotta De Carlo, Shingo Kuwata, Evelyn Regar, Fabian Nietlispach, Adolfo Ferrero, Alberto Weber, Francesco Maisano

**Affiliations:** 1 Department of Cardiovascular Surgery, University Hospital Zürich, Rämistrasse 100, 8091 Zürich, Switzerland; 2 Department of Cardiology, University Hospital Zürich, Rämistrasse 100, 8091 Zürich, Switzerland

**Keywords:** aortic stenosis, TAVI, surgical risk

## Abstract

Over the last few years, treatment of severe symptomatic aortic stenosis in high-risk patients has drastically changed to adopt a less-invasive approach. Transcatheter aortic valve implantation (TAVI) has been developed as a very reproducible and safe procedure, as shown in many trials. When compared to surgery, TAVI has produced superior, or at least comparable, results, and thus a trend to broaden treatment indications to lower-risk patients has erupted as a natural consequence, even though there is a lack of long-term evidence. In this review, we summarize and underline aspects that still remain unanswered that are compulsory if we want to enhance our understanding of this disease.

## Introduction and historical background

Aortic stenosis (AS) is the most common heart valve disease worldwide, ranging from 2–4% in patients older than 75 years of age ^1, 2^. Progressive valve calcification as a degenerative and aging process has been found to be the most frequent cause in first world countries, followed by congenital alterations (such as bicuspid leaflets) and rheumatic disease ^1^. As a result of this progressive calcific degeneration, symptoms might remain hidden in a latent asymptomatic stage of the disease until signs of left ventricular (LV) impairment appear. These include exertional dyspnea (shortness of breath), angina-like symptoms (chest pain), dizziness, and syncopes and rhythm alterations up to sudden death. The latter are unlikely to be found, whereas shortness of breath and angina-like symptoms are very common among symptomatic patients. The natural history of this pathology, being a degenerative and progressive disease, is unstoppable, and sooner or later a valve replacement is mandatory in order to prevent irreversible hemodynamic changes. Many studies have shown advantages of valve replacement over medical treatment ^2– 4^.

In order to prevent irreversible changes, such as LV remodeling, an invasive treatment is mandatory. In the past, many patients were not referred for surgery because of high surgical risk, mainly owing to advanced age, LV impairment, and concomitant comorbidities ^4^. Thus, a lot of patients were managed conservatively. In order to fill this gap comprising almost 33% of patients ^4^, a less-invasive alternative treatment was developed. The transcatheter aortic valve implantation (TAVI) procedure was first described in 2002 ^5^. Fifteen years later, the TAVI procedure has become standard treatment for selected patients. Over the last few years, with enhanced diagnostic techniques, our understanding of this pathology has changed considerably. Today, we address native aortic valve disease with a multidisciplinary heart team that includes interventional cardiologists, cardiac surgeons, anesthesiologists, and echocardiographers to give patients an adequate therapy. In addition, even if a previous surgically implanted bioprosthesis happens to malfunction, TAVI appears to be an optimal solution (TAVI-in-valve) in high surgical risk populations.

The more we have learned about the disease, the more questions have emerged, which is one of the main topics of this article. We will focus on recent and current advances addressing AS over the last few years and the way it has changed the management of the disease itself. In addition, we will underline factors of considerable importance that remain incompletely understood and that are compulsory if we want to address this disease properly.

## Current advances

### Diagnostic management

Thanks to the introduction of specialized diagnostic tools, we can now have a better understanding of the whole valve’s anatomy. Through the standardized use of three-dimensional (3D) echocardiographic as well as multi-slice computed tomography (MSCT) preprocedural assessment before intervention, a rapid and accurate visualization and analysis of the aortic valve is possible, facilitating the pre-operative planning of aortic valve procedures ^6^.

Almost every big trial was performed without 3D echocardiography ^7, 8^ and moreover without computed tomography (CT) scans and the 3D measurements. In order to assess preprocedural measurements, an electrocardiogram (ECG)-triggered CT scan of the heart and the whole aorta, including femoral and subclavian arteries, is performed. Not only can aortic annulus size be studied using MSCT but also leaflet and annulus calcification. The latter can be removed during surgery but, if present, might stand in the way of TAVI. Other important characteristics to be taken into account are distances between the annulus and the coronary ostia that could differ from standard and could result in ostial occlusion after implantation. On the other hand, left ventricular outflow tract (LVOT) and proportions of the ascending aorta are mandatory to achieve a precise and safe implantation. Moreover, the peripheral access site and the descending aorta can be evaluated for anomalies such as major calcification, stenosis, and other factors that could hinder the procedure ^9^. MSCT is now an essential tool in terms of access site evaluation, prosthesis sizing, and reducing the paravalvular leakage and risk of complications ^6^.

### Results

Many trials have already shown efficacy and even better outcomes than surgery in selected high surgical risk patients ^7, 8, 10, 11^. Since then, many patients have been treated with TAVI and similar results were achieved. With the passing of the years and since positive results were observed, there has been a trend to broaden treatment indications ^8, 12, 13^. The PARTNER 1 trial randomized high-risk and non-operable patients to either TAVI or surgical aortic valve replacement (SAVR) ^7^. Results regarding survival, stroke, bleeding complications, valve hemodynamics, and many more were comparable in both groups. All-cause mortality (TAVI versus SAVR) was found in 24.2% and in 26.8% at 1 year and in 67.8% and in 62.4% at 5 years, respectively. Periprocedural stroke appeared more frequently in the TAVI group, but 5-year results demonstrated that this difference equalized over time (15.9% versus 14.7%). In terms of valve hemodynamics and, more specifically, valve deterioration, there was no particular difference between the two groups ^7^.

**Figure 1.**
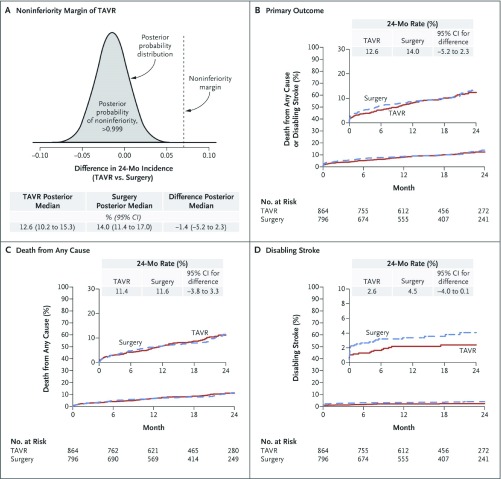
SURTAVI Outcomes. ( **a**) Confirmation of the non-inferiority margin for TAVI. ( **b**) Time-to-event for death from any cause or disabling stroke. ( **c**) Time-to-death from any cause. ( **d**) Time-to-disabling stroke. From Reardon *et al.*, 2017 ^12^. Reprinted with permission from Massachusetts Medical Society.

**Figure 2.**
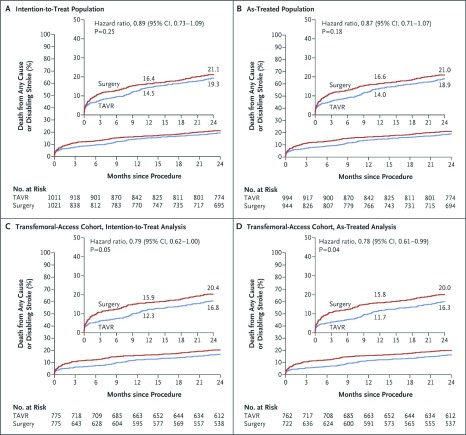
PARTNER 2 Trial Outcomes. ( **a**) Time-to-event for death from any cause or disabling stroke in the intention-to-treat population, ( **b**) in the as-treated population, ( **c**) in the transfemoral-access group in the intention to treat, ( **d**) and in the as-treated analysis. From Leon *et al.*, 2016 ^8^. Reprinted with permission from Massachusetts Medical Society.

Intermediate-risk patients were enrolled in new trials (SURTAVI and PARTNER 2) and they were consequently treated with the transcatheter approach. Results were not as superior as in high-risk patients, but they were comparable to SAVR ^8, 12^ (See Figure 1 and Figure 2, respectively). The main differences between the PARTNER 1 and 2 trials were the inclusion of lower-risk patients (mean STS 12% versus 5.8%), new available devices (SAPIEN versus SAPIEN XT) with enhanced and smoother features, and, above all, advanced operator experience. In terms of results, the PARTNER 2 trial showed non-inferiority compared to SAVR. All-cause mortality rates were similar with 12.3% versus 12.9% at 1 year, and 16.7% versus 18% at 2 years in the TAVI and SAVR groups, respectively. Disabling stroke appeared at a similar frequency at 1 and 2 years and hemodynamics remained stable also.

According to current European guidelines of treatment, early therapy should be performed in all symptomatic severe AS (Class IB). The decision of whether to choose TAVI over SAVR in patients at increased risk should be assessed by the Heart Team (Class IB), taking into account individual characteristics, comorbidities, and technical aspects (Class IC) ^14^ (see Table 1).

**Table 1.** Guidelines of treatment for aortic stenosis (AS).

	Recommendation	Evidence grade
**American guidelines** **2017 (AHA/ACC)**	TAVI or SAVR in symptomatic severe AS + high surgical risk	IA
	TAVI for symptomatic severe AS **AND** a prohibitive risk for SAVR with a post- TAVI survival of >12 months	IA
	TAVI as a reasonable alternative to SAVR for severe symptomatic AS and an **intermediate surgical risk**	IIa, B–R
	Percutaneous aortic balloon dilation may be considered as a bridge to SAVR or TAVI for severe symptomatic AS	IIb, C
	A heart team is mandatory for patients in whom TAVI or high surgical risk SAVR is an option	IC
**European guidelines** **2017 (ESC/EACTS)**	TAVI in symptomatic severe AS patients who are not suitable for SAVR assessed by a heart team	IB
	TAVI or SAVR in patients at increased risk (STS score or EuroSCORE II ≥4% or log EuroSCORE ≥10%) or when other risk factors (i.e. frailty, porcelain aorta, sequelae of chest radiation) are present	IB
	TAVI only in centers with both cardiac surgery AND cardiology with a structured collaboration (Heart Team) TAVI or SAVR in severe symptomatic AS based on individual evaluation including clinical characteristics, anatomical and technical aspects, and cardiac conditions in addition to AS	IC IC

Abbreviations: AHA/ACC, American College of Cardiology/American Heart Association; EACTS, European Association for Cardio-Thoracic Surgery; ESC, European Society of Cardiology; SAVR, surgical aortic valve replacement; STS, Society of Thoracic Surgeons; TAVI, transcatheter aortic valve implantation.

Actuarial American guidelines of treatment recommend TAVI in severe symptomatic AS patients with a prohibitive surgical risk and a predicted post-procedural survival greater than 1 year (Class IA). Both SAVR and TAVI are recommended for severe symptomatic AS and high surgical risk. The decision to perform one or the other depends on patient-specific procedural risks and more (Class IA). In patients felt to be at an intermediate risk for surgery, TAVI appears to be a reasonable alternative to SAVR in symptomatic individuals, depending on patient-specific characteristics (Class IIA and level of evidence C-LD) ^15^ (see Table 1).

Thyregod *et al.* reported similar results in low-risk patients compared to SAVR, but no significant superiority could be shown regarding the primary outcome of study, namely the composite of death from any cause, stroke, or myocardial infarction ^16^. PARTNER 3 and UK TAVI are ongoing trials that will reveal further information about this topic.

TAVI can be easily performed through different access sites, such as transfemoral, transsubclavian, transaortic, transcarotid, or transapical. The safest as well as the most adopted and favorite access point is transfemoral owing to similar or even better results in terms of mortality compared to surgery. On the other side, transthoracic implantation was linked to comparable or worse results than surgery in terms of outcome ^8^. Blackstone and colleagues found, in a propensity match analysis from the PARTNER 1 trial, a greater periprocedural morbidity and mortality and also longer hospital length of stay in the transapical group than in the matched transfemoral population ^17^.

Hemodynamics are better in those patients in short- and long-term follow-up than in surgery; in other words, mean gradients are lower and valve areas are bigger after TAVI than after SAVR ^8, 12^.

Furthermore, valve durability has been observed in up to 5 years of follow-up, but it remains an issue over this timeframe. Hence, and until consistent evidence is documented, we need to be careful and select suitable patients.

### Complications

Probably one of the most important complications after TAVI is the occurrence of cerebrovascular events (CVEs), which include stroke as well as transient ischemic attack (TIA). These have been found, according to actuarial data, in several trials in 0.6–10%, and their appearance has been linked with an impaired clinical outcome with an increased mortality up to tenfold compared to patients without stroke ^18^. The Bern TAVI Registry, the Swiss TAVI Registry, and the France TAVI Registry showed similar incidence rates (3.6%, 3.3%, and 3.6%, respectively) ^18^. As mentioned above, the occurrence of CVEs is related to higher mortality rates. Eggebrecht *et al.* showed in a meta-analysis of around 10,000 patients an increased mortality after stroke (>3.5-fold) ^19^. Similar results could be found in the German TAVI Registry with a fivefold increase in the in-hospital mortality rate ^20^. Differences between first- and new-generation devices have prompted a decrease in CVE appearance. Initial data from the CoreValve trial suggested stroke rates at 30 days and 1 year in 2.3% and 4.3%, respectively, whereas these rates decreased in the SURTAVI study to 1.2% and 2.2%. Similar results were found in PARTNER 1 and 2 with diminishing stroke rates from 5.0% to 3.2% at 30 days and 7.8% to 5.0% at 1 year ^7, 8, 12, 21, 22^. Even though the appearance of this important complication is low, its clinical impact is devastating. In this context, and to prevent CVEs, it has been suggested that a predilation prior to implantation should be abandoned. Hamm and colleagues did not find a reduction of CVEs in a retrospective analysis comparing balloon-expandable prostheses with or without predilation. However, procedure and fluoroscopy time and subsequent radiation were shorter without predilation ^23^. In a small series trial, the investigators were not able to show any difference in stroke rate with or without predilation. Nonetheless, they found a higher number of ischemic brain lesions analyzed by diffusion-weighted magnetic resonance imaging (DW-MRI) after TAVI without predilation ^24^. Silent cerebral ischemic lesions with no particular short-term neurological deficit are present in around 70–100% of all TAVI patients, and they have been suggested to be linked with poor neurological outcome ^25, 26^.

Another method to reduce CVE incidence would be the use of embolic protection devices (EPDs) during TAVI. The SENTINEL trial studied the use of one of these EPDs. However, even though it was a safe procedure, there was no statistical significance in major adverse cardiovascular and cerebrovascular events (MACCEs) and stroke reduction ^27^. In another trial, the DEFLECT III, the authors were able to prove reduction in the primary in-hospital safety endpoint (death, stroke, major bleeding, major vascular complication, and acute kidney injury) and also freedom from new ischemic brain lesions was greater, assessed with DW-MRI ^28^. Whether we want to apply EPDs generally must be investigated in detail.

Moderate to severe paravalvular aortic regurgitation (AR), although a natural consequence, is an unwanted complication after TAVI, with an incidence of 12.2% seen in the PARTNER trial; this is contrary to SAVR, where this condition is uncommon and has been found in only 0.9% ^29^. Moderate to severe paravalvular AR is strongly associated with lower short- and long-term survival rates compared to those patients with no to mild paravalvular AR after TAVI ^7, 8^. It has been proved that the appearance of paravalvular leak (PVL) has a relationship with annulus undersizing as well as severe annular calcification and incorrect implantation of the prosthesis ^30^. Nowadays, the incidence of a hemodynamic relevant PVL should be expected to be lower, thanks to a much broader spectrum of devices (sizes, models, etc.) and thanks to new imaging methods (MSCT analysis) ^6, 31^. The main differences between first-generation devices with regard to relevant PVL must be noted. Adams *et al*. reported an incidence of 14.6% at 1 year with the self-expandable valve ^32^, whereas Reardon and colleagues found relevant PVL in 5.3% of cases at 1 year ^12^. As expected, a reduction between first- and second-generation balloon-expandable valves was also found with a 1-year incidence of PVL ≥3+ in 6.8% and 3.4%, respectively. These findings can be explained through smoother device design and, of course, thanks to enhanced operator experience ^7, 8, 12, 32^.

A couple of techniques have been described to suppress PVL, such as balloon post dilatation, valve-in-valve implantation, the so-called snare technique, and interventional closure depending on PVL pathology ^30^ in addition to surgical correction. Interventional closure has been shown to be a safe and effective alternative with comparable results to surgical correction in selected patients ^33– 35^. When taking into account younger trials with novel devices, we should be able to observe a decreasing trend of relevant PVL appearance.

Major vascular complication originates mainly from the puncture area, namely the groin in transfemoral approaches, and is also influenced by antithrombotic/antiplatelet therapy. Through the introduction of smaller, less harmful catheters and sheaths, the complication rate has decreased substantially ^31^. In addition, closure devices have been developed to prevent such events (i.e. access site bleeding) in order to minimize complications and longer hospitalizations.

Pacemaker implantation is required in 5.9 to 25.8% after TAVI, due to atrioventricular dissociation, depending on the type of the implanted valve. It has been linked to higher hospitalization time, re-hospitalization, and higher mortality in short-term follow-up ^36^.

### Antiplatelet and anticoagulation therapy

Current guidelines regarding antithrombotic therapy after TAVI recommend the use of antiplatelet drugs and oral anticoagulants depending on the concomitant comorbidities, even though there has been no randomized evaluation that has focused on optimal strategies ^37, 38^. The fact that up to 30% of patients undergoing TAVI have an indication for oral anticoagulation stands in the way of proper antithrombotic therapy ^39^.

Ongoing trials (ARTE, AUREA, ATLANTIS, and GALILEO) will hopefully provide us with more evidence on which agent is most suitable. The Aspirin Versus Aspirin + ClopidogRel Following Transcatheter Aortic Valve Implantation: the ARTE Trial (ARTE) is seeking to compare the use of aspirin and clopidogrel as a dual antiplatelet therapy and aspirin alone in preventing major ischemic events, like ischemic stroke and myocardial infarction or death, without raising the risk of major bleeding events at 12 months.

Investigating the incidence of major vascular events (hemorrhagic or ischemic) 3 months after TAVI and consequently initiation with antithrombotic treatment with either oral anticoagulation or dual antiplatelet therapy is the principal purpose of the Dual Antiplatelet Therapy Versus Oral Anticoagulation for a Short Time to Prevent Cerebral Embolism After TAVI (AUREA) trial. Magnetic resonance imaging (MRI) will be performed 3 months post-replacement in both arms to evaluate cerebral changes.

In ATLANTIS (Anti-Thrombotic Strategy to Lower All Cardiovascular and Neurologic Ischemic and Hemorrhagic Events After Trans-Aortic Valve Implantation for Aortic Stenosis), the investigators formulate the hypotheses that apixaban (an anti-factor-XA inhibitor) is superior to standard-of-care therapies to prevent cardiovascular events after TAVI. Primary outcomes comprise death, myocardial infarction, stroke, systemic embolism, intracardiac or bioprosthesic thrombus, any episode of deep vein thrombosis or pulmonary embolism, and life-threatening, disabling, or major bleeding at 1-year follow-up ^40^.

With a similar primary outcome to ATLANTIS, a total of around 1,500 patients after successful TAVI and without atrial fibrillation will be enrolled in the Global Study Comparing a rivAroxaban-based Antithrombotic Strategy to an antipLatelet-based Strategy After Transcatheter aortIc vaLve rEplacement to Optimize Clinical Outcomes (GALILEO) study. An anticoagulation therapy with aspirin and rivaroxaban will be compared to standard-of-care dual antiplatelet-based therapy after TAVI for 3 months. Afterwards, monotherapy with either rivaroxaban or aspirin will be continued ^41^.

### Prosthetic valve thrombosis decreased leaflet motion and prosthetic valvular endocarditis

Occurring in less than 1% of cases, symptomatic transcatheter aortic-valve thrombosis is a very rare condition that might be associated with decreased leaflet motion in its initial stages. Several coexisting factors have been theoretically postulated to be associated with prosthetic valve thrombosis (PVT): (i) prothrombotic factors, (ii) the metallic structure as well as an (iii) inadequate expansion of the prosthesis, (iv) an insufficient contact to the aortic wall that might delay endothelialization, and (v) the reminiscent native leaflets that could create blood flow stagnation.

Asymptomatic decreased leaflet motion has been assessed and confirmed through 4-dimensional CT scan and transesophageal echocardiography (TOE) but not with transthoracic echocardiography (TTE). CT scan was felt to be more accurate in diagnosing PVT than TEE. Almost 75% of the patients in whom PVT was found were successfully treated with any form of anticoagulation ^42– 44^. Larger trials are mandatory in order to confirm risk factors as well as to establish standard PVT management.

Because of the less-invasive nature of transcatheter valve interventions, one would be forgiven for thinking that the incidence of prosthetic valve endocarditis (PVE) is lower than in surgery. In fact, early PVE tends to appear in between 0.3% and 3.4% according to actuarial data, wherein the incidence of surgical PVE is around 0.3% and 1.2% per patient year with a mortality of >20% ^44^. In the PARTNER trial, surgical and transcatheter PVE were present at a similar rate ^7^. The available literature suggests on one hand that PVE is associated with risk factors such as diabetes, chronic kidney disease, immunosuppression, and recurrent infections ^43^; however, on the other hand, male sex, low implantation site, ≥ moderate PVL, valve-in-valve, and vascular complications have also been suggested to be associated with higher risks of PVE. Periprocedural factors could also support endocarditis (i.e. failure on antibiotic prophylaxis and dental procedures, lower cath-lab sterilization conditions). Reported cases treated conservatively with antibiotics resulted in acceptable outcomes with an in-hospital mortality lower than SAVR (11% and 22%, respectively) ^44, 45^.

### Structural valve failure

In terms of structural valve failure (SVF) in surgical valves, Pibarot *et al*. reported a rate of failure in patients older than 70 years of age of <10% at 10 years ^2^. In younger patients (<40 years of age), however, the incidence of structural failure is around 20–30%. The incidence of SVF with respect to TAVI is low with no reported valve deterioration or need to replace at 5 years in the PARTNER trial ^7^. Smaller trials also suggest similar failure rates ^2^. Owing to the natural high-risk patient characteristics and their lower expected survival, a proper evaluation with longer-term follow-up similar to surgery is of utter importance if we expect to broaden indications to lower-risk patients.

### Valve Academic Research Consortium-2 criteria

In 2011, the first edition of The Valve Academic Research Consortium (VARC) criteria manuscript was published, aiming to get a consensus in selecting appropriate clinical endpoints reflecting not only device- but also patient- and procedure-related safety and effectiveness, as well as standardizing definitions for single and composite clinical endpoints for TAVI in clinical trials. The VARC-2 criteria were described subsequently to update definitions and selection of TAVI endpoints for actuarial and future trials and to enhance risk stratification and case selection ^46^ (see Table 2).

**Table 2.** Valve Academic Research Consortium (VARC)-2 criteria.

*Risk stratification*	STS score and EuroSCORE should include other factors (frailty, porcelain aorta, severe liver disease, hostile chest, severe pulmonary hypertension) as well as co-existing conditions (i.e. anemia, dementia)
*Immediate procedural mortality*	Death within 72 hours after TAVI
*Myocardial infarction (periprocedural* *<72 hours and spontaneous* *>72 hours)*	Systematic collection of biomarkers prior to TAVI, within 12–24 hours after TAVI, at 24 hours thereafter, and at 72 hours or at discharge. If still elevated, daily until decline shows up.
*Stroke; TIA*	Disabling and non-disabling using the modified Rankin scale (mRS)
*and detailed stroke etiology*	Ischemic, hemorrhagic, and undetermined
*Access site*	Detailed information regarding access site and pre-planned vascular closure technique
*Vascular complications*	Access (i.e. iliac rupture) and non-access site-related (ascending aorta dissection or rupture, etc.)
*Closure device failure*	Not anymore within vascular complications; considered as part of the TAVI procedure, unless clinical consequences are documented
*Bleeding complications*	As a result of overt bleeding and not only as a result of blood transfusion
*Acute kidney injury (AKI)*	From 72 hours to 7 days according to AKIN guidelines
*Assessment of conduction* *disturbances*	New and/or worsened conduction disturbances; up to 72 hours’ continuous monitoring
*Echocardiography parameters*	Valve function: position, morphology, evaluation of left and right ventricle size and function; patients’ own initial post-implant measurements should be used as a reference; hemodynamics initially: one flow-dependent (i.e. mean gradient) and one flow- independent (i.e. effective orifice area [EOA]) criterion, if discordance then Doppler velocity index (DVI) should be calculated. Indexed EOA should be assessed in other to unmask prosthesis-patient-mismatch (PPM).
*Follow-up assessments*	Valve imaging and documentation of changes in morphology
*Quality of life (QOL) assessment*	Heart-failure-specific measure (The Minnesota Living with Heart Failure Questionnaire [MLHF] and/or the Kansas City Cardiomyopathy Questionnaire [KCCQ]) and a more generic measure between 30 days and 5 years
*Composite endpoints*	Time-related valve safety: a combination of valve dysfunction, endocarditis, and thrombotic complications of the prosthesis; valve performance through the indexed EOA

Abbreviations: AKIN, Acute Kidney Injury Network; STS, Society of Thoracic Surgeons; TAVI, transcatheter aortic valve implantation; VARC, Valve Academic Research Consortium.

Follow-up assessment, as mentioned above, should document valve imaging and changes in morphology, comparing this measurements with patients’ own post-implant imaging. An alteration in hemodynamics, such as a reduction in the Doppler velocity index (DVI) >0.1–0.13, a decrease in the effective orifice area (EOA) of more than 0.3–0.4 cm², or an increasing mean gradient >10 mmHg, suggest a change in valve function.

### New devices

Initial studies were performed with first-generation devices, which have been enhanced over the last few years. We now have a wide variety of different prostheses and sizes that help us make the best decision and individual selection. It has been proposed that new implantation tools have a potential to reduce complications (PVL, pacemaker implantation, stroke, and vascular complications) at long-term follow-up. The design of these prostheses has been reshaped in order to achieve a smoother, more precise structure that adapts better to native anatomical tissues ^23^.

## Conclusion

Many questions still remain unanswered. We know that TAVI is a very feasible procedure that, in terms of outcomes in high-risk patients, is even better than surgery. Results in intermediate- and lower-risk patients are comparable to surgery, but we still need more evidence if we want to broaden indications. Complications if they appear are manageable; nevertheless, the impact of them, in terms of both valve failure and long-term results, still remains an issue.

The future belongs to TAVI, indeed, but contrary to surgery, where large analyses exist, we do not really know about long-term durability, and, above all, we do not know if changes will be the same in patients at intermediate or lower risk.

## Abbreviations

3D, three-dimensional; AR, aortic regurgitation; AS, aortic stenosis; CT, computed tomography; CVE, cerebrovascular event; DVI, Doppler velocity index; DW-MRI, diffusion-weighted magnetic resonance imaging; ECG, electrocardiogram; EOA, effective orifice area; EPD, embolic protection device; LV, left ventricular; LVOT, left ventricular outflow tract; MSCT, multi-slice computed tomography; PVE, prosthetic valve endocarditis; PVL, paravalvular leak; PVT, prosthetic valve thrombosis; SAVR, surgical aortic valve replacement; SVF, structural valve failure; TAVI, transcatheter aortic valve implantation; TIA, transient ischemic attack; TOE, transesophageal echocardiography; TTE, transthoracic echocardiography; VARC, The Valve Academic Research Consortium.
